# Electroosmotic Pumps with Frits Synthesized from Potassium Silicate

**DOI:** 10.1371/journal.pone.0144065

**Published:** 2015-12-02

**Authors:** Sara Nilsson, Per G. Erlandsson, Nathaniel D. Robinson

**Affiliations:** Transport and Separations Group, Department of Physics, Chemistry and Biology, Linköping University, Linköping, Sweden; Irvine, UNITED STATES

## Abstract

Electroosmotic pumps employing silica frits synthesized from potassium silicate as a stationary phase show strong electroosmotic flow velocity and resistance to pressure-driven flow. We characterize these pumps and measure an electroosmotic mobility of 2.5×10^-8^ m^2^/V s and hydrodynamic resistance per unit length of 70 ×10^17^ Pa s/m^4^ with a standard deviation of less than 2% even when varying the amount of water used in the potassium silicate mixture. Furthermore, we demonstrate the simple integration of these pumps into a proof-of-concept PDMS lab-on-a-chip device fabricated from a 3D-printed template.

## Introduction

Without on-chip pumps, sophisticated lab-on-a-chip (LOC) devices often require multiple (and problematic) external tubing connections and external pumps, resulting in complex systems that depend on extensive off-chip machinery. Electroosmotic pumps (EOPs) can be integrated into chips and provide connector-free fluid control with a uniform (plug-flow) velocity profile [1, 2] without pulses, reducing sample band broadening. EOPs also have a small footprint, and only require electrical connections for effective flow control [3]. Although long slender capillaries are the typical conceptual model used when describing electrokinetic transport, packed silica spheres [4, 5] or silica spheres sintered to form a silica frit with μm-scale porosity [6] act as EOPs when a potential difference is applied. Furthermore, they also resist pressure driven flow, for example when the potential is removed [7].

Electroosmotic (EO) flow, generated by the influence of an electric field on the mobile ions found near the wall of a container, can only effectively drive flow in vessels with diameters less than a few hundred micrometers [8]. The EO mobility, the flow velocity per applied field strength, depends only on the surface chemistry of the wall and the solution being pumped [9]. The velocity of pressure-driven (Poiseuille) flow, on the other hand, decreases exponentially as the radius decreases, U_hyd_ ∝ r^2^. For a circular capillary, the pressure-driven flow rate scales as Q_hyd_ = ΔP/R_hyd_ ∝ r^4^, whereas the EO-driven flow rate scales as Q_EO_ = U_EO_A = μ_EO_Eπr^2^ ∝ r^2^, where U_EO_ is the EO-driven flow velocity [m/s], Q is the volumetric flow rate [m^3^/s], R_hyd_ is the hydrodynamic resistance [Pa s/m^3^], μ_EO_ is the EO mobility [m^2^/V s] and E is electric field strength [V/m] [5, 10]. Thus, to maximize EO flow while minimizing pressure-driven flow and maintaining the overall cross-sectional area available for flow, the smallest diameters achievable yield the best performance.

The channels need not be straight to produce an effective pump. A channel packed with μm-sized silica spheres [4, 5] or containing a silica frit [6] can also generate EO flow. Potassium silicate (KSi) solutions are able to self-assemble into silica frits with tortuous channels that are useful in High-Performance Liquid Chromatography (HPLC) [11], but can also be used as EOPs. Previous work has demonstrated good pressure-generation with KSi frit based EOPs with poor reproducibility [12]. Here, we study this process and highlight a probable cause for the variation in performance reported previously. The result is a robust process for producing frits from potassium silicate (KSiFs) inside 100 μm diameter fused silica (FS) capillaries. EOPs using KSiFs were characterized in terms of their μ_EO_ and R_hyd_ with buffer solutions typically used in biochemistry. We propose that stable KSiFs can be fabricated reproducibly inside FS capillaries and yield effective EO pumps, and that microscopic defects in the frit, easily visible upon careful inspection in a microscope, are the likely cause of the poor reproducibility in pump performance reported previously [12].

## Materials and Method

KSiFs were produced in untreated FS capillaries (Genetec AB) with 100 μm inner- and 365 μm outer-diameter (including the external polyimide coating). Unless otherwise indicated, chemicals were supplied from Sigma Aldrich. Prior to filling the 150 mm long capillaries with a KSi mixture, they were washed with (in sequence) methanol, aqueous 1 M NaOH, and aqueous 1 M HCl. The capillaries were rinsed with DI-water before and after each wash solution, and finally dried with N_2_. The capillaries were thereafter filled with a 9:1 mixture of Kasil 1 (8.3 wt% K_2_O and 20.8 wt% SiO_2_ in H_2_O, PQCorp) and formamide (≥99%). Rubber septa were attached to the ends of the capillaries to retain the KSi mixture inside the capillaries before placing the capillaries in a ventilated oven (Heraeus UT6P) at 90°C for 1 hour, converting the KSi mixture to a continuous silica frit. Each capillary was cut into 20 mm long sections with a ceramic cutting stone (Genetec AB).

In addition to the standard recipe (procedure described above), KSiFs were also made with two mixtures containing additional water, in volume ratios of 18:2:2 and 18:2:1 of Kasil 1: formamide: water.

After production, all capillary sections were treated equally and characterized as follows: Steady-state ionic resistance (R_i_ = V/I) was measured by insertion of a 20 mm capillary between two wells in a polystyrene dish (Nunc 8-well rectangular dish, Thermo Scienific) modified by cutting a notch between two adjacent wells. The notch was sealed with silicone, and each well filled with 10 ml of phosphate-buffered saline (PBS) mixed 1:9 with 100 mM NaCl and adjusted with 1 M NaOH to pH 7.4 (hereafter referred to as HCE, High Concentration Electrolyte). Applying a potential between Pt-electrodes (0.5 mm diameter Pt replacement wires, Sigma Aldrich) placed in each well and measuring the current allowed the resistance to be calculated. The system is designed so that the potential drop occurs almost entirely over the capillary or frit being tested, which we confirmed by measuring the potential drop over an empty capillary using a Ag/AgCl pseudo-reference electrode. The drop was measured to be 148.5 V when 150 V was applied between the Pt electrodes, which means the (combined) potential drop at (both) the electrodes was about 1.5 V. The same drop (1.5 V) was measured with 50 V applied between the electrodes.

Transient R_i_ was measured with the method described above with the exception that the salt concentration at the ends of the capillary differed. One well was filled with HCE and the other with 10 ml of HCE diluted 10% by adding DI-water (LCE). The polarity of the applied potential was alternated and the current through the system measured as a function of time [13, 14].

Hydrodynamic resistance (R_hyd_) for 20 mm long capillary sections was measured with a LabSmith microfluidic system by driving solution at a constant flow rate (Q_hyd_) with a syringe pump (LabSmith SPS01, 4 μl barrel) through sections and measuring the pressure drop between the ends (ΔP), R_hyd_ = ΔP /Q_hyd_. One end of the capillary section was open to ambient atmosphere while the other was inserted into a microfluidic interconnect tee (306–203, LabSmith), together with a pressure sensor (uPS0800-C360-10, LabSmith) and tubing connected to the syringe pump. The pump delivered DI-water at a constant flow rate of 0.35 nl/sec for at least 40 min while the pressure at the intersection was recorded. R_hyd_ of the empty 100 μm FS capillaries could not be measured with this setup since the resulting pressure was smaller than the minimum pressure the sensor was capable of reporting accurately, and was instead estimated from the capillary dimension and fluid properties (assuming Poiseuille flow).

**Fig 1:**
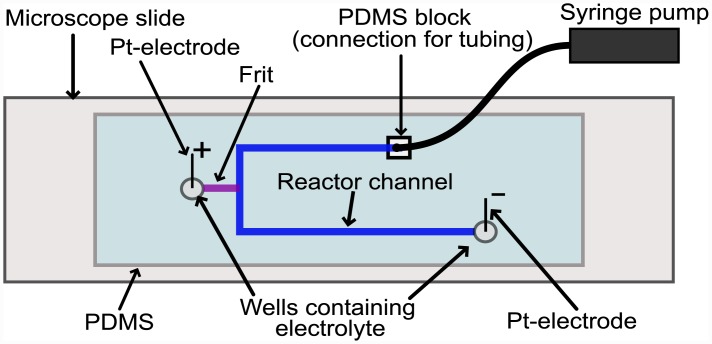
Schematic of a microfluidic device used for testing the EO pump. A potential applied between the Pt electrodes (labelled “+” and “-”) in their respective open wells creates an electric field over the frit (purple), pumping sample from the anodic well (left of the frit) into the main fluidic system (dark blue).

In order to demonstrate their viability for real-world applications, a KSiF EOP was integrated into a simple lab-on-a-chip system simulating, for example, substance-delivery to a micro-bioreactor with a continuous flow of cell media, see Fig 1. Using a syringe-based 3D-printer (Fab@Home Model 3, Seraph Robotics), a sacrificial blend of 75 wt% polyethylene glycol, PEG2000 (2000 g/mol, Alfa Aesar), and 25 wt% propylene carbonate, PC (99.5%, Acros Organics) [15], a nearly cylindrical template (syringe-tip diameter: 0.84 mm) was deposited on a microscope slide (76x26 mm^2^, VWR). This template will later be removed to form the main channel of a fluidic device that could be used as a simple microbioreactor. The sacrificial material was annealed at 40°C for 60 min, removing the PC by evaporation. Before inserting one end of the KSiF into the printed PEG template, it was protected by filling it with PEG200 (Alfa Aesar), which has a lower molecular weight (200 g/mol) and viscosity than PEG2000 as well as lower crystallization temperature, both of which facilitate its removal. A drop of PEG2000 was added to the other end of the KSiF with a pipette tip, creating a template for a well. The system was covered with a 10:1 PDMS mixture (polydimethylsiloxane and curing agent, Sylgard^™^ Dow Corning), and the PDMS cured at 21°C overnight, then at 75°C in an oven for 15 minutes. Wells (approx. 3 mm in diameter) were punched in the PDMS at the ends of the printed channels and the free end of the frit, then the whole system was immersed in a 70°C water bath, removing the PEG blend from the 1 mm wide and 0.7 mm high main channel. Filling the system with water and heating it while applying a potential to generate EOF removed any remaining PEG from the KSiF. Using a syringe-pump, LCE was introduced into the main channel whereas the well next to the KSiF was filled with HCE. Pt-electrodes were placed in the open wells according to the schematic in Fig 1 and potentials were applied and current measured as previously described in the previous paragraph.

## Results and Discussion

**Fig 2:**
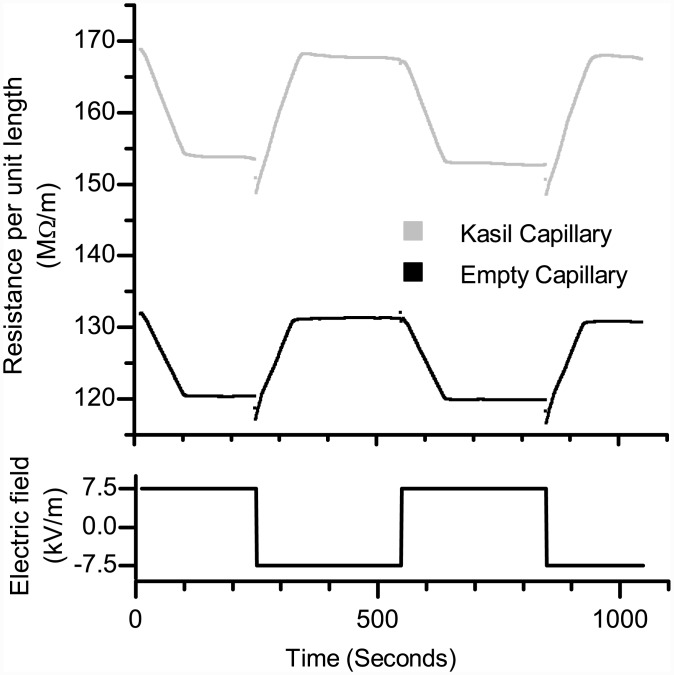
Electroosmotic transient ionic resistance measurement. Top: Ionic resistance per unit length R_i_/L as a function of time recorded while pumping high and low ionic strength solutions through L = 20 mm long and 100 μm i.d. capillary sections that were either empty or filled with a silica frit synthesized from potassium silicate. Bottom: The applied electric field V/L driving the flow. The similarity between the time required for the resistance of the empty and filled capillaries to rise (and fall) indicates that the electroosmotic (EO) mobility of the empty and filled capillaries are nearly the same.

Results from the transient ionic resistance measurement (see S1–S4 Figs and S1 Dataset) were used to estimate electroosmotic velocities. As the interface between the HCE and LCE traverses the capillary, the average resistance in the capillary/frit changes nearly linearly with time, see Fig 2. The rate-of-change of the resistance was used to calculate the average effective EO velocity U_EO_, and ultimately the electroosmotic (EO) mobility, μ_EO_ = U_EO_/E [13, 14]. KSiFs demonstrated an EO mobility μ_EO_ = 2.5×10^−8^ m^2^/V s, at most 10% less than that of an empty fused silica capillary, based on the average fluid velocity recorded neglecting the increase in the distance the fluid travels because of the tortuosity of the pores in the frit. The zeta potential ζ of the KSi frit, based on this mobility, was calculated to be -36 mV using ζ = μ_EO_ * η/(ε_r_ε_0_) where the solution viscosity η = 1.01 cP [16] and the relative dielectric constant ε_r_ = 80 [17]. This zeta potential matches that reported previously [18].

**Fig 3:**
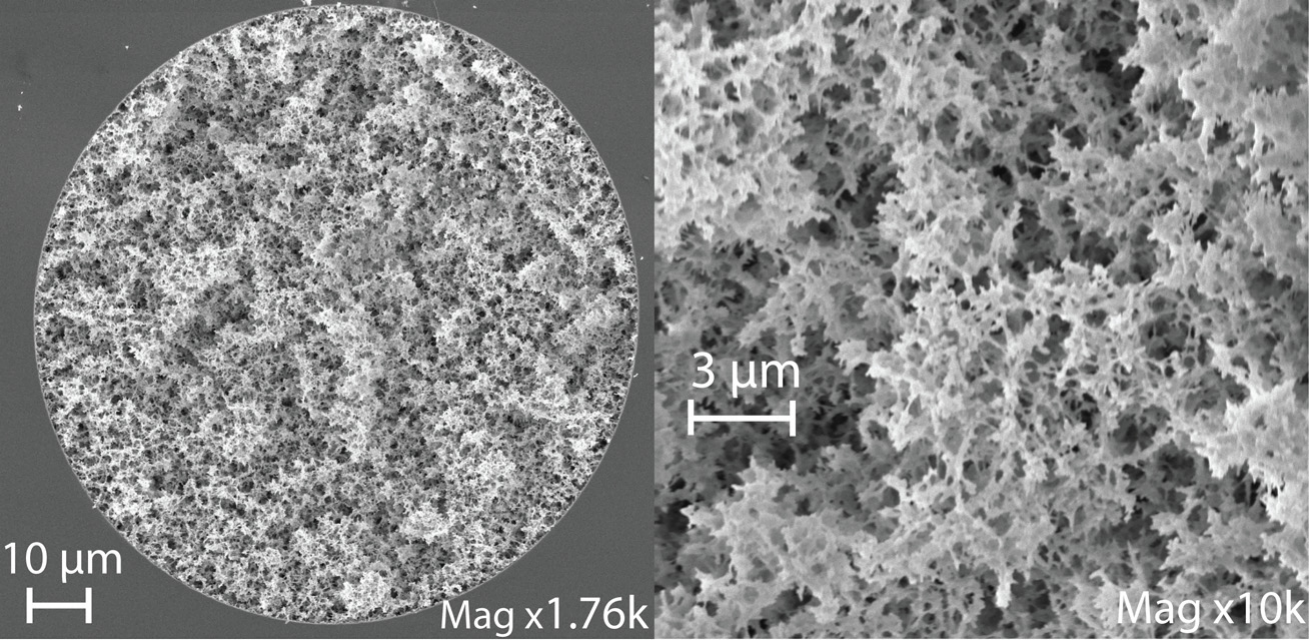
SEM images of a cross-section of FS capillary containing silica produced with extra water (18:2:2 K:FA:H_2_O). The length scale of the microstructure is similar to the estimated equivalent capillary diameter of 0.85 μm.

We measured the hydrodynamic resistance (R_hyd_) of the KSiF to be over four orders of magnitude larger than the predicted value for the empty capillary, see Table 1, S5 Fig and S1 Dataset. Given the relative resistance and flow rates, one can calculate the number and size of multiple parallel small cylindrical channels that would result in the same R_hyd_ and R_i_ as the KSiFs. On average, a 100 μm diameter KSiF performs roughly equivalently to 11200 parallel channels each having a diameter of 0.85 μm. The latter is notably the same order of magnitude as the length scale of the structures observed in SEM images, see Fig 3, and two orders of magnitude smaller than the empty capillary. Compared to an empty capillary, the KSiFs have 17600 times higher R_hyd_ and show a 6% lower μ_EO_. The volumetric flow rate through the KSiF is 27% lower than for an empty capillary, approximately 1.2 nl/sec for 150 V across a 20 mm capillary, see Table 1, due to a combination of the 20% reduction in capillary volume caused by the frit and the reduced μ_EO_.

**Table 1:** Comparison between electroosmotic properties of an empty FS capillary and silica frits produced with varying water content. R_i_/L values were measured on capillaries filled with a 9:1 mix of 100 mM NaCl and PBS with pH 7.4. Standard deviation (last column) compares data from all frits, even though the recipe was intentionally varied, demonstrating the favorable reproducibility of devices made with the method described here.

Volume ratio of K:FA:H_2_O in KSi-mixture	Empty	18:2:0	18:2:1	18:2:2	Std dev KSi
Mass frac. K_2_O + SiO_2_ in KSi-mixture	N/A	0.266	0.255	0.246	
KSiF Stationary Phase Fraction	0	0.214	0.196	0.183	
R_i_/L [MΩ/m]	124	157	153	151	2
EO Mobility [10^-8^ m^2^/V s]	2.70	2.53	2.59	2.53	0.02
R_hyd_/L [10^17^ Pa s/m^4^]	0.004	71.8	68.6	70.7	1.3
Num. equiv. Capillaries	1	10900	11200	11600	400
Equivalent diameter [μm]	100	0.85	0.85	0.84	0.01
EOF rate at 7.5 kV/m [nl/sec]	1.59	1.17	1.24	1.22	0.04

Comparing the R_i_ from steady-state ionic resistance measurements of empty capillaries and KSiF yields an estimate of the volume fraction of the mobile phase in the KSiF devices. The KSiFs have an average stationary volume fraction of 0.2. Increasing the water content in the KSi-mixture decreases both the weight fraction of KSi in the mixture and the stationary phase fraction in the final frit, see Table 1.

Varying the water content produced frits that appeared identical during inspection under an optical microscope. SEM images (LEO 1550 Gemini, field emission) were taken to estimate the characteristic length of the frit structure, see Fig 3. The different amounts of water in the KSi-mixtures resulted in different microstructures in the frits produced, (compare Fig 3 to S6 and S7 Figs); a higher water content appears to result in thinner silica structures and smaller characteristic lengths. The pump characteristics, EO mobility and R_hyd_, for capillaries produced with varying water content do not differ significantly, but the stationary phase fraction and structural morphology do. To quantify this, a standard deviation has been estimated (and reported in the last column of Table 1) using the values obtained with frits of varying water content (columns 3–5). These values include the variation caused by the (intentionally) varied recipe and any (unintentional) irreproducibility of the fabrication and measurement processes.

Visual inspection of the KSiF cross-sections under a stereo microscope revealed holes, about 15 μm in diameter, in the segments cut from the ends of the 150 mm capillaries. Segments from the middle of the capillaries did not contain this defect, but up to 60% of the original 150 mm capillary was observed to contain such a vacancy running along the length of the capillary at its center. Varying the KSi mixture and curing procedure proved ineffective in removing these defects. All results presented here were obtained from sections of the capillary that appeared homogenous and free of defects. The sections with holes showed large variation in performance, as described in the supplementary information (see S8 Fig and S1 Table), which may explain the “poor reproducibility” of the pressure generated by KSiF-based EOPs reported by Wang et al. [12]. When an inspection is included, this method for producing KSiFs is robust and reproducible enough to give standard deviations of 3% or less of the reported values even when (intentionally) varying water content in the recipe, see Table 1.

**Fig 4:**
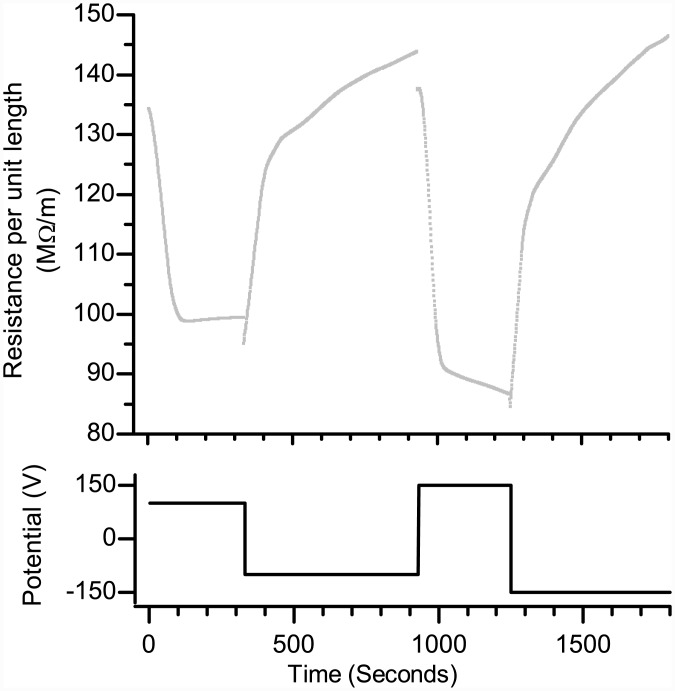
Top: Ionic resistance per unit length R_i_/L recorded while alternately pumping high (potential > 0) and low (potential < 0) ionic strength solutions through L = 20 mm long and 100 μm i.d. sections of a capillary filled with a silica frit integrated into a PDMS device as a function of time. Bottom: The applied potential V (+/- 100 and +/- 150 V) driving the flow.

Although the primary intention of this study was to characterize the silica frits in a well-defined geometry (the capillary), we demonstrated the use of the frit-filled capillary in a PDMS microfluidic system molded onto a 3D printed sacrificial template, shown schematically in Fig 1. Fig 4 shows the result of a transient R_i_ measurement for a PDMS/glass device with an integrated KSiF. The μ_EO_ and R_i_ are approximately the same as measured for the frits characterized in Fig 2, demonstrating that PEG was removed and that the EOP still works after being enclosed in the PDMS. However, evaporation of water from the relatively small reservoirs in the LOC device caused the ionic strength of the HCE to increase during this measurement, resulting in a decreased resistance at the start of, and a further decrease over the course of, the experiment compared to the frits characterized in the system with large wells shown in Fig 2. Furthermore, HCE “contaminates” the larger fluidic channel, causing a slow increase in the measured resistance as it is slowly removed when pumping from the channel to the reservoir (electric field < 0, e.g., between 400 and 900 seconds).

## Conclusion

In this work, we verify that KSiFs, previously used primarily to contain packing material like silica spheres within liquid chromatography columns, can be used to provide the small-dimension medium required for reliable and effective electroosmotic pumps that are resistant to pressure-driven flow. We report a mean μ_EO_ of 2.5×10^−8^ m^2^/V s with a standard deviation of less than 1% even when varying the amount of water used in the mixture. This, in turn, enables on-chip EO pumps in low-cost microfluidic devices made with, for example, PDMS to drive effective EOF and resist pressure-driven flow. Since EO pumps drive flow and manipulate samples in lab-on-a-chip devices with only electronic, rather than fluidic or pneumatic, connections, the pumps reduce the number of high-cost and failure-prone fluidic couplings between sophisticated chips and external controller devices as the fluidic contacts can be replaced with more reliable and inexpensive electronic contacts. Electronic control also enables the use of on-chip sensors and logic (transistors) to control the pumps. Finally, the incorporation of a KSiF into a simple fluidic device made from a 3D-printed template demonstrates that this technology can be used to create the small “diameter” passages required to generate EO flow in a device made by a fabrication system that is otherwise unable to generate features with sufficiently small dimensions. This, in combination with printable electrode materials that eliminate water electrolysis and its side-effects [13], help make highly-functional and largely autonomous 3D-printed lab-on-a-chip systems possible.

## Supporting Information

S1 Dataset Raw data for ionic resistance measurement and hydrodynamic measurement. (XLSX)

S1 Fig Ionic resistance of frit made with 18:2:0 mixture. Top: Ionic resistance per unit length R_i_/L as a function of time recorded while pumping high and low ionic strength solutions through a L = 20.3 mm long and 100 μm i.d. capillary section filled with a 18:2:0 silica frit. Bottom: The electric field V/L driving the flow as ±150 volt is applied across the system.
(PDF)

S2 Fig Ionic resistance of frit made with 18:2:2 mixture. Top: Ionic resistance per unit length R_i_/L as a function of time recorded while pumping high and low ionic strength solutions through a L = 19.4 mm long and 100 μm i.d. capillary section filled with a 18:2:2 frit. Bottom: The electric field V/L driving the flow as ±150 volt is applied across the system.
(PDF)

S3 Fig Ionic resistance of frit made with 18:2:1 mixture. Top: Ionic resistance per unit length R_i_/L as a function of time recorded while pumping high and low ionic strength solutions through a L = 19.6 mm long and 100 μm i.d. capillary section filled with a 18:2:1 frit. Bottom: The electric field V/L driving the flow as ±150 volt is applied across the system.
(PDF)

S4 Fig Ionic resistance of an empty capillary. Top: Ionic resistance per unit length Ri/L as a function of time recorded while pumping high and low ionic strength solutions through a L = 19.2 mm long and 100 μm i.d. empty capillary section. Bottom: The electric field V/L driving the flow as ±150 volt is applied across the system.
(PDF)

S5 Fig Hydrodynamic resistance of frits produced from various mixtures. Pressure per unit length across capillary sections with a pressure driven flow of 21 nl/sec. Comparison between frits produced with KSi mixtures with different water content: 18:2:0, 18:2:2 and 18:2:1 (K:FA:H_2_O), as indicated in the legend.
(PDF)

S6 Fig SEM image of a cross-section of a frit made with the standard Kasil recipe. The frit was made with the standard Kasil recipe (no extra water). This segment came from the middle of a 150-mm capillary, where no large defect is visible (in contrast to the segment shown in S7 Fig).
(TIF)

S7 Fig SEM image (higher magnification) of a cross-section of a frit made with the standard Kasil recipe. SEM close-up of the same cross-section shown in S6 Fig.
(TIF)

S8 Fig SEM image of a cross-section of a frit made with the standard Kasil recipe showing the “hole” defect. The frit was made with the standard Kasil recipe (no extra water). This segment came from the end of a 150-mm capillary. The hole visible at the center of the frit has a large influence on the frit’s hydrodynamic resistance.
(TIF)

S1 Table Pumping performance for frit-filled capillary segments with and without holes. Electroosmotic and hydrodynamic performance of an empty capillary compared with frit segments (standard Kasil recipe with additional water) taken from the end of a 15-cm capillary (with a defect like that shown in S8 Fig) and from the middle of the same capillary (without a defect, as shown in S6 Fig).
(DOCX)
