# Supplementary figures and images for: Electroosmotic Pumps with Frits Synthesized from Potassium Silicate
Source: PLoS One. 2015 Dec 2;10(12):e0144065. doi: 10.1371/journal.pone.0144065 (PMC4668094; doi:10.1371/journal.pone.0144065)

# Ionic Resistance: Frit 18:2:0

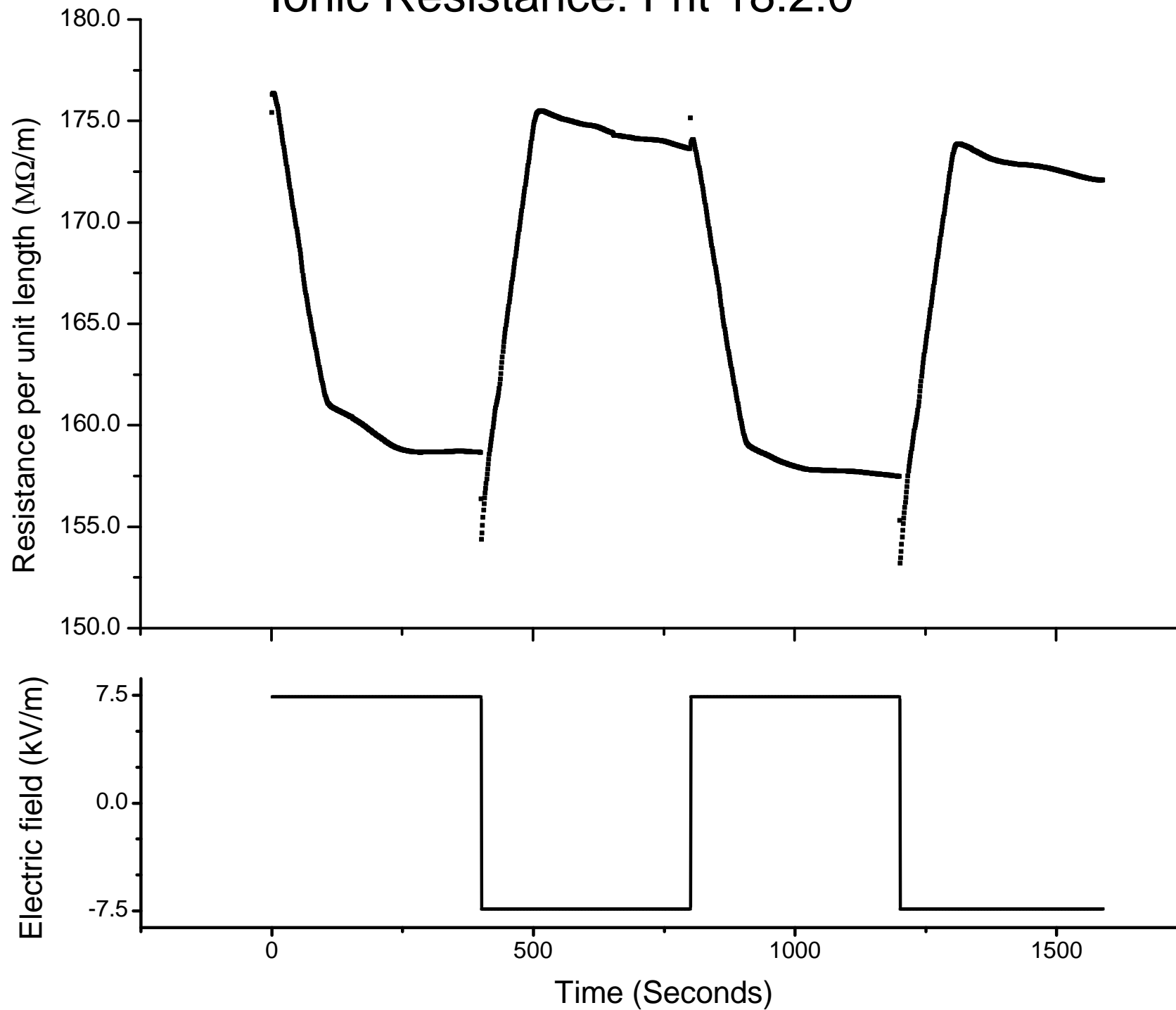

Supplement: S1 Fig — Top: Ionic resistance per unit length Ri/L as a function of time recorded while pumping high and low ionic strength solutions through a L = 20.3 mm long and 100 μm i.d. capillary section filled with a 18:2:0 silica frit. Bottom: The electric field V/L driving the flow as ±150 volt is applied across the system. (PDF) [file pone.0144065.s002.pdf]

# Ionic Resistance: Frit 18:2:2

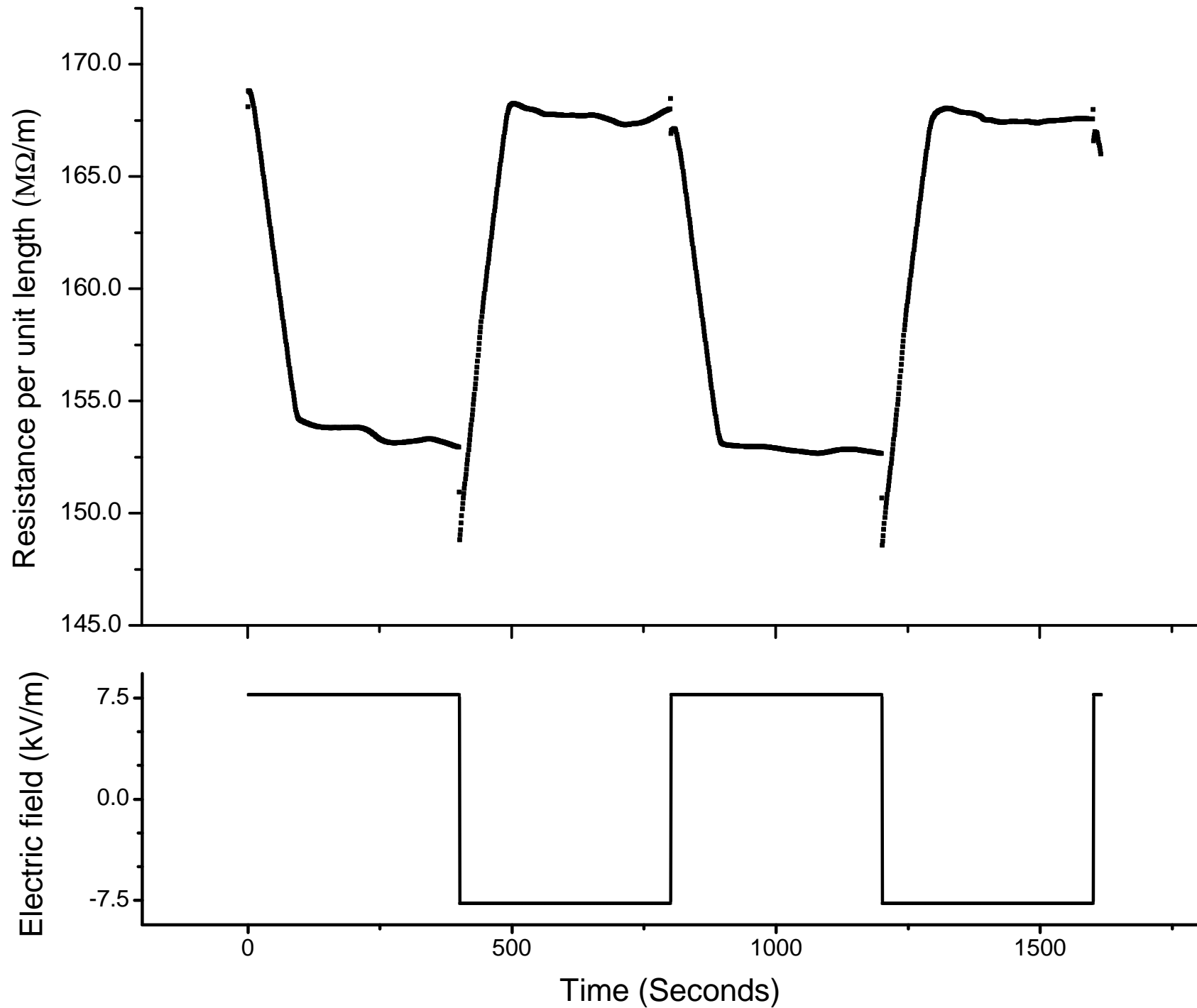

Supplement: S2 Fig — Top: Ionic resistance per unit length Ri/L as a function of time recorded while pumping high and low ionic strength solutions through a L = 19.4 mm long and 100 μm i.d. capillary section filled with a 18:2:2 frit. Bottom: The electric field V/L driving the flow as ±150 volt is applied across the system. (PDF) [file pone.0144065.s003.pdf]

# Ionic Resistance: Frit 18:2:1

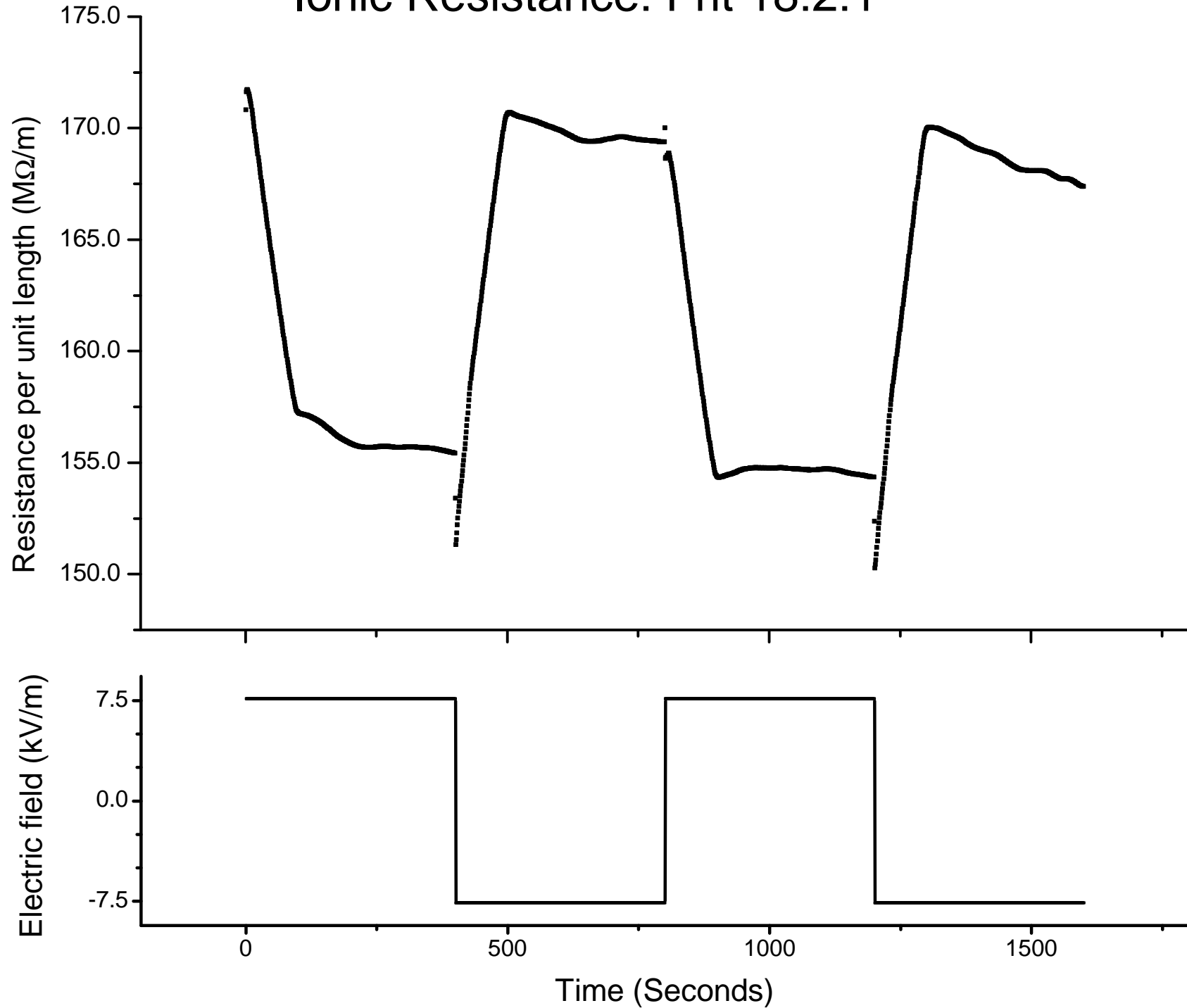

Supplement: S3 Fig — Top: Ionic resistance per unit length Ri/L as a function of time recorded while pumping high and low ionic strength solutions through a L = 19.6 mm long and 100 μm i.d. capillary section filled with a 18:2:1 frit. Bottom: The electric field V/L driving the flow as ±150 volt is applied across the system. (PDF) [file pone.0144065.s004.pdf]

# Ionic Resistance: Empty Capillary

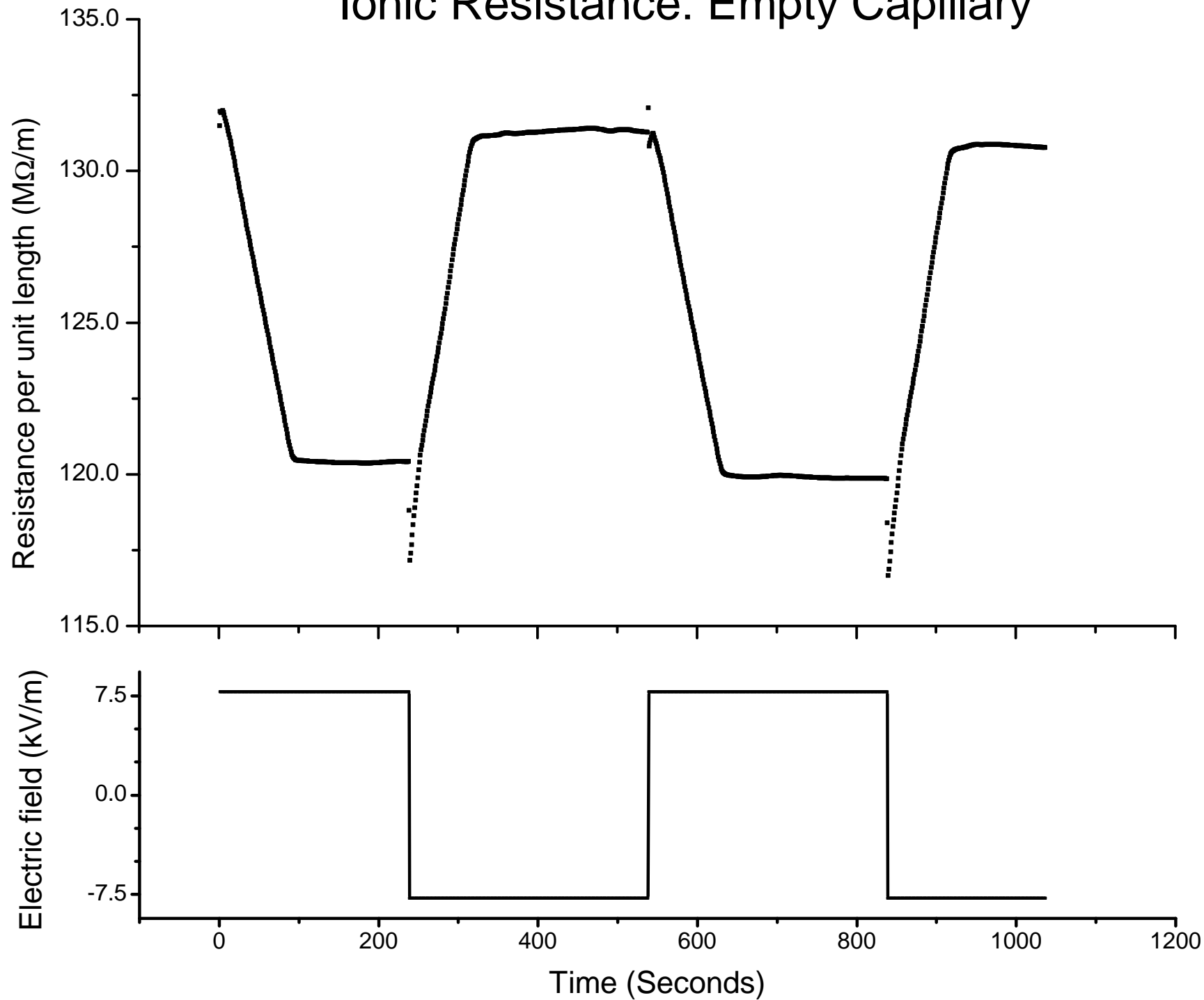

Supplement: S4 Fig — Top: Ionic resistance per unit length Ri/L as a function of time recorded while pumping high and low ionic strength solutions through a L = 19.2 mm long and 100 μm i.d. empty capillary section. Bottom: The electric field V/L driving the flow as ±150 volt is applied across the system. (PDF) [file pone.0144065.s005.pdf]

# Hydrodynamic resistance measurement

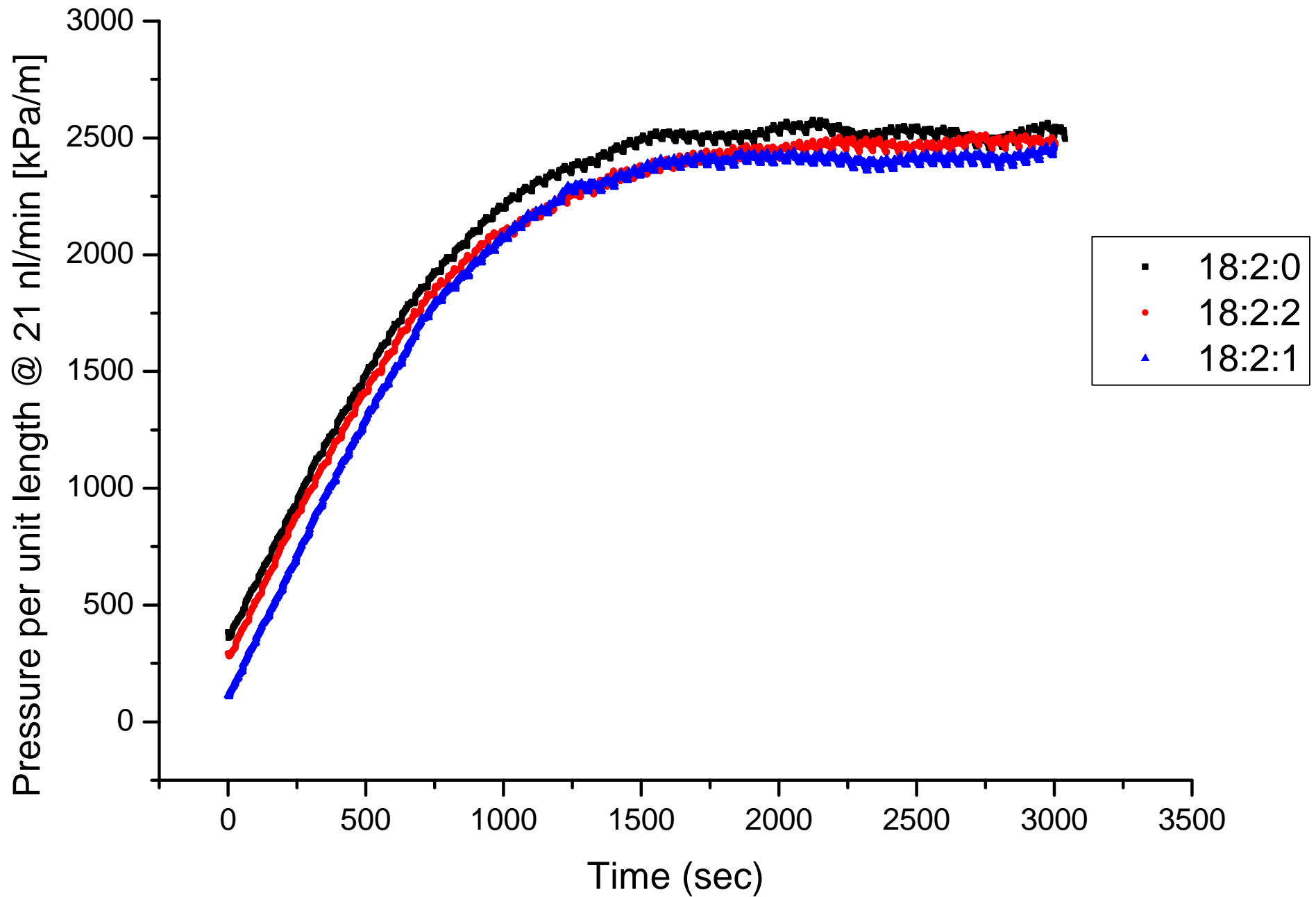

Supplement: S5 Fig — Pressure per unit length across capillary sections with a pressure driven flow of 21 nl/sec. Comparison between frits produced with KSi mixtures with different water content: 18:2:0, 18:2:2 and 18:2:1 (K:FA:H2O), as indicated in the legend. (PDF) [file pone.0144065.s006.pdf]
