# Supplementary material for: Electroosmotic Pumps with Frits Synthesized from Potassium Silicate
Source: PLoS One. 2015 Dec 2;10(12):e0144065. doi: 10.1371/journal.pone.0144065 (PMC4668094; doi:10.1371/journal.pone.0144065)
Supplement: S1 Table — Electroosmotic and hydrodynamic performance of an empty capillary compared with frit segments (standard Kasil recipe with additional water) taken from the end of a 15-cm capillary (with a defect like that shown in S8 Fig) and from the middle of the same capillary (without a defect, as shown in S6 Fig). (DOCX) [file pone.0144065.s010.docx]

| **Volume ratio K:FA:H_2_O in KSi-mixture** | **Empty capillary** | **Center segment** | **End segment** |
| --- | --- | --- | --- |
| Weight Fraction K_2_O + SiO_2_ in KSi-mixture | N/A | 0.266 | 0.266 |
| KSiF Stationary Phase Fraction | 0 | 0.214 | 0.315 |
| R_i_/L [kΩ/mm] | 125 | 157 | 177 |
| EO Mobility [10^-8^ m^2^/V·s] | 2.67 | 2.51 | 2.27 |
| R_hyd_/L [10^17^ Pa·s/m^4^] | 0.004 | 71.4 | 23.0 |
| Equivalent Capillaries | 1 | 9820 | 2450 |
| Equivalent Ø [µm] | 100 | 0.87 | 1.64 |
| EOF at 7500 V/m [nl/sec] | 1.64 | 1.16 | 0.87 |
